# Remarkable Anticancer Activity of *Teucrium polium* on Hepatocellular Carcinogenic Rats

**DOI:** 10.1155/2014/726724

**Published:** 2014-08-13

**Authors:** Ariyo Movahedi, Rusliza Basir, Asmah Rahmat, Mohammad Charaffedine, Fauziah Othman

**Affiliations:** ^1^Department of Nutrition and Dietetics, Faculty of Medicine, Universiti Putra Malaysia, Malaysia; ^2^Department of Human Anatomy, Faculty of Medicine, Universiti Putra Malaysia, 43400 Serdang, Selangor, Malaysia; ^3^Diseased Tissue Pathology Laboratory, Tyre, Lebanon

## Abstract

The term cancer has been concomitant with despair, agony, and dreadful death. Like many other diseases, herbal therapy has been used to prevent or suppress cancer. The present study investigated the capability of the decoction of *Teucrium polium* L. from Lamiaceae family to protect liver cells against hepatocellular carcinoma in carcinogenesis-induced animal model. After 28 weeks of treatment with decoction of *Teucrium polium* L., serum biochemical markers including ALT, AST, AFP, GGT, ALP, HCY, TNF-*α*, *α*2MG, and CBG have been regulated auspiciously. Total antioxidant status also has been increased intensely. Liver lesion score in treated group was lessened and glucocorticoid activity has been intensified significantly. In conclusion, *Teucrium polium* L. decoction might inhibit or suppress liver cancer development.

## 1. Introduction

Hepatocellular carcinoma (HCC), the predominant form of liver cancer, is the sixth most common cancer and the third most frequent cause of cancer-related death worldwide [1, 2]. Patients diagnosed with HCC have a poor prognosis due to the violent nature of the disease [3]. On the other hand, surgical resection or local extirpation therapy is effective only at an early stage of HCC. Hence, any preventive or suppressive method would be vital. Like other diseases, complementary medicine has been commonly considered in cancer. Herbal medicine has been widely used to cure or prevent diseases since ages. Many of the herbs contain phytochemicals, antioxidants, flavonoids, and dietary fibers which have shown anticancer properties [4, 5].* Teucrium polium* L. (Lamiaceae), known popularly as Golden/Felty germander, is a subshrub and is native to southwest Asia and the Mediterranean region [6, 7]. Its flowers and its leaves are used both in cooking and for medicinal purposes, particularly for the treatment of stomach ailments.* T. polium* has been long used in Iran commonly as decoctions or infusions for its diuretic, antipyretic, diaphoretic, antispasmodic, tonic, anti-inflammatory, antihypertensive analgesic, antibacterial, and antidiabetic effects [8–12]. An infusion of the leaves and flowers of the plant is also consumed as a refreshing beverage [13]. In recent years,* T. polium* has been tested and showed beneficial effects on nonalcoholic steatohepatitis [14] and it has been shown to be considered as an effective and safe chemosensitizer agent for cancer therapy [15].* In vitro* study on its aqueous extract has shown that it can effectively inhibit oxidative processes and has substantial antioxidant activity [16]. Based on some isolated compounds from this plant such as diterpenoids, flavonoids, iridoids, sterols, and terpenoids [11, 17], it might have cytotoxic activity and antitumor properties. As herbal decoctions are one of the major techniques in herbal medicine and could be drunk as a part of daily dietary intake, in the present study, the effect of* T. polium* decoction has been investigated on hepatocellular carcinoma in animal model.

## 2. Materials and Methods

### 2.1. Plant Material and Preparation of the* T. polium* Decoction

High quality dried Iranian* T. polium* (Herbarium number US00050655) leaves were purchased from certified herbal marketing in Tehran, Iran (ParsiTeb Co.), and shipped to Malaysia. Dried* T. polium* leaves were weighed and washed 3 times with tap water and then were put into a 10-liter beaker. For each 100 g of dried leaves, 4000 mL of distilled water was added. Then the mixture was heated up to 70°C to reduce the water content to 1000 mL through evaporation. After these phases, the residues were filtered. The liquids were chilled and kept in the fridge at 4°C in hygienic bottles until being used.

### 2.2. Force-Feed Calculation

As a neutraceutical study, instead of different concentration which is more common in pharmaceutical studies, the usual decoction technique and one concentration has been used. The Institute of Medicine determined that an adequate intake (AI) for men is roughly 3 liters (about 13 cups) of total beverages a day. The AI for women is 2.2 liters (about 9 cups) of total beverages a day [18]. The average daily water intake of Sprague-Dawley rats in chronic studies is about 15 mL/100 g body weight [19, 20]. As average standard human body weight is 65 kg (71 for male and 61 for female) [21], it is possible to drink and recommend two cups of any kind of herbal tea. Hence, to calculate the best amount of force feeding of the decoction of the herb, the following calculation was used: 2 of 13 cups per day are equal to 460 mL →460 ÷ 65≅7 mL/kg body weight →0.7 mL/100 g body weight.

### 2.3. Animals and Experimental Protocols

The present study was designed as a preclinical study [22]. The protocol of the rat hepatocarcinogenesis in this study was according to Solt and Farber method [23]. Forty male rats, 8 ± 1 weeks old, with average weight 243.1 ± 6.7 g, were divided into groups of three and maintained at 60 ± 5% relative humidity and 22 ± 1°C with a 12 h light/dark cycle. Allrats had free access to the standard rat food pellet based on AIN-76A [24] and tap water during the study. Hepatocarcinoma was induced in 30 of the rats by single intraperitoneal injection of 200 mg/kg diethyl nitrosamine (DEN) dissolved in corn oil and then followed by a cancer promotion period of 2 weeks on food, which was mixed with 2-acetylaminofluorene (0.02% AAF) as a promoter of hepatocarcinogenesis without partial hepatectomy to promote hepatocarcinogenesis. The rats were then left for 2 weeks. A group of 10 rats served as normal group with no DEN injection or hepatocarcinogenesis promoter diet. After the cancer initiation period, the leftover rats were weighed again and divided randomly into two groups with no significant differences in their weight. Both control and* T. polium* groups were allowed free access to AIN76 and water* ad libitum* for 28 weeks, but rats in* T. polium* group were force-fed 0.7 mL/100 g body weight/day of* T. polium* decoction.

### 2.4. Chemicals and Biochemical Analyses

Alpha-fetoprotein tumor marker (AFP), tumor necrosis factor-alpha (TNF-*α*), homocysteine (HCY), corticosteroid binding globulin (CBG), and alpha-2-macroglobulin (*α*2MG) were analyzed using standard commercial ELISA kit (Cusabio Biotech, China). Gamma-glutamyl transpeptidase (GGT) was tested by using Colorimetric Assay Kit (BioVision, USA). Alanine aminotransferase (ALT/SGPT), aspartate aminotransferase (AST/SGOT), alkaline phosphatase (ALP), and total antioxidant status (TAS) were analyzed by Chemical Pathology Lab at FMHS, UPM, using Roche Cobas C-311 analyzer.

### 2.5. Decoction Characteristic Analysis

Decoction total phenolic content was evaluated with Folin-Ciocalteu's phenol reagent [25, 26] and colorimetric aluminum chloride method was used for flavonoid determination [25]. The antioxidant activities of the decoction were determined based on its ability to scavenge 2,2-diphenyl-1-picrylhydrazyl (DPPH) radicals, based on method of Brand Williams and coworkers (1995) [27]. Butylated hydroxyanisole (BHA), rutin, and *α*-tocopherol were used as positive control for synthetic and natural antioxidant, respectively.Antioxidant activity was reported as IC_50_, defined by the concentration of samples required (mg/mL) to scavenge 50% of the free radicals. Based on the literature, rutin and apigenin are two active compounds of* T. polium* and have been analyzed by high-performance liquid chromatography (HPLC) using services from ChromaDex, Inc., CA, USA.

### 2.6. Histopathological Examinations

Half of the liver tissue of each sample was fixed in 10% formalin and then the paraffin blocks were prepared. The sections from blocks were stained with hematoxylin-eosin. The histopathological evaluations were performed blindly by an expert pathologist using a scoring system. The lesion scoring was done according to the revised method of Batts and Ludwig by Stevens et al. [28, 29]. The rest of the livers were saved in −80°C for liver glucocorticoid receptor analysis. Fluorescent* in situ* hybridization (FISH) was used to analyze glucocorticoid receptor RNA activity by QuantiGene ViewRNA ISH Tissue 2-Plex Assay kit (Affymetrix Inc., USA). For positive control, ACTB and GAPD were used as housekeeping genes. The frozen tissues were cut using rotary cryomicrotome (Leica 1850 UV) at 4–8 microns and pasted on a slide for FISH test. Slides were observed under confocal laser microscope (Olympus FV10, Japan). In order to view fluorescent activity, the following filters have been used. For Fast Red Substrate Cy3/TRITC (filter set: Excitation: 530 ± 20 nm, Emission: 590 ± 20 nm, Dichroic: 562 nm), for Fast Blue Substrate, Cy7-B/Alexa 750 (custom filter set: Excitation: 630 ± 20 nm, Emission: 775 ± 25 nm, Dichroic: 750 nm), and for DAPI filter set (Excitation: 387/11 nm, Emission: 447/60 nm) were used.

### 2.7. Statistical Analyses

Data were expressed as mean ± SEM. Statistical differences between normal, treated, and control groups were determined using one-way repeated measures analysis of variance (ANOVA) followed by Duncan's multiple range as post hoc test. Differences between groups were considered significantly different when *P* value was less than 0.05.

## 3. Results

Total phenolic and flavonoid content of the used decoction were 2.635 ± 0.001 mg/mL and 0.081 ± 0.002 mg/mL, respectively. DPPH scavenging activity was shown in Table 1. HPLC results of the used* T. polium* of this study failed to find both rutin and apigenin as active compounds. Therefore, the benefits of this herb would be due to other phenolic components.

Throughout the intervention some of the rats in both groups died. Almost one-third of the rats in the control group died, while* T. polium* treated group showed a significantly lower mortality rate (*P* < 0.05) and only 8.3% of them died. After 28 weeks of treatment, based on the present outcomes, rats in the control group showed higher but no significant weight gain as compared to* T. polium* treated group, 490.14 ± 18.77 versus 461.1 ± 15.0 (*P* > 0.05). In spite of the significantly higher liver weight among control groups, 12.25 ± 0.34 versus 10.50 ± 0.49 g (*P* < 0.05), no significant liver weight ratio was observed, 2.47 ± 0.06 versus 2.28 ± 0.10 (*P* > 0.05).

The effect of* T. polium* on serum liver function markers as compared to control has been illustrated in Table 2. Both normal group and* T. polium* treated group showed significantly lower serum ALP (*P* < 0.05) as compared to the control group. Similar results have also been found for AST, ALT, HCY, and *α*2MG (*P* < 0.05). Despite the lower level of AST/ALT ratio in the normal group, no significant difference was found between groups (*P* > 0.05). Significantly higher level of CBG was found in* T. polium* treated group and the lowest value was detected in normal group (*P* < 0.05). Although* T. polium* group showed significantly higher levels of TNF-*α*, *α*2MG, AFP, and GGT as compared to normal (*P* < 0.05), all these markers were significantly lower than control group (*P* < 0.05). The extremely lowest level of TAS was observed in the control group (*P* < 0.01), while* T. polium* treated group showed a considerably higher TAS level than normal group (*P* < 0.05).

**Figure 1:**
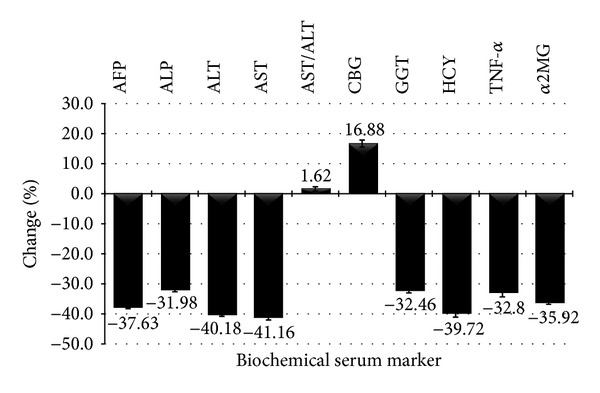
Percentage change of different serum biochemical markers in* T. polium* group as compared to the control group.

As Figure 1 shows, after 28 weeks of treatment with* T. polium* decoction, most of serum liver function and cancer markers have improved and dropped significantly as compared to the control. AFP, ALP, ALT, and AST were reduced significantly by 37.63 ± 1.29%, 31.98 ± 1.02%, 40.18 ± 0.69%, and 41.16 ± 0.51%, respectively (*P* < 0.05). AST/ALT ratio has slightly increased by 1.62 ± 1.24% (*P* > 0.05). CBG has improved and increased significantly by 16.88 ± 0.86% (*P* < 0.05). Moreover,* T. polium* decoction was able to raise TAS drastically by 861.47 ± 0.38%. Serum GGT, HCY, TNF-*α*, and *α*2MG in* T. polium* treated group decreased significantly by 32.46 ± 0.25%, 39.72 ± 0.57%, 32.8 ± 1.04%, and 35.92 ± 0.78% (*P* < 0.05).

### 3.1. Histopathological Findings

**Figure 2:**
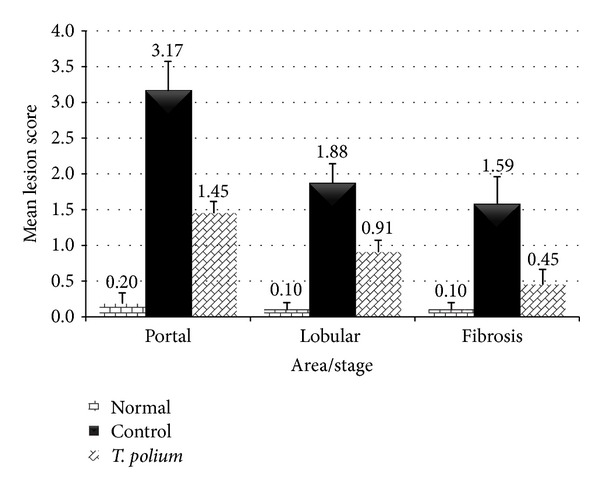
Effect of* T. polium* on mean lesion score of rats' liver tissue as compared to the control.

**Figure 3:**
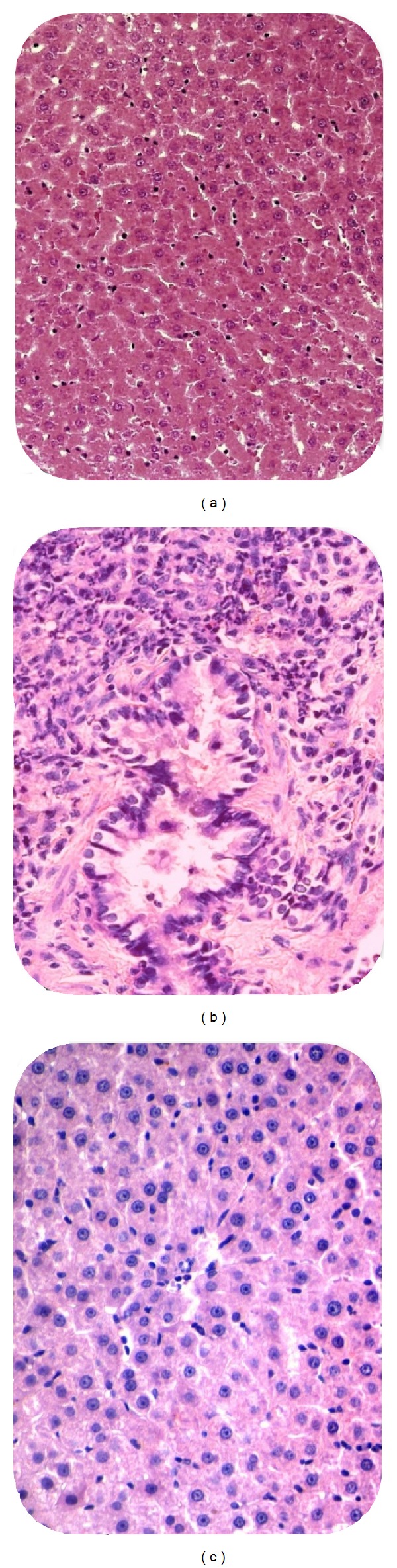
Light micrograph of liver cell in different groups. (a) Normal liver cell at the lobular region of normal group, lesion score: 0. (b) HCC in control group, lesion score: 4. (c) Hepatitis in* T. polium*, lesion score: 2. H&E, ×400.

Lesion score of different groups is shown in Figure 2. Based on the present findings, rats in control group showed a significantly higher lesion score in both portal and lobular region as well as fibrosis stage compared to all other groups (*P* < 0.05). Perceptibly, normal group had the lowest lesion score (*P* < 0.05) due to their health status.* T. polium* treated group showed significantly lower lesion score in all sites as compared to the control group (*P* < 0.05) which was illustrated in Figure 3 as well.

## 4. Discussion

In this project, the control group had the highest mortality rate, which was expected. Gross histology of* T. polium* group showed no hepatic nodules in this group, unlike the control ones. Differences in the mortality rate with other studies could possibly be explained by either the used dosage of DEN or duration of the study [30]. Unfortunately, there were no comparable data about mortality rate of* T. polium* in a cancer study; however, the mortality rate of both control and treated groups was rational and similar to other studies [31]. The relationship between HCC and body weight is still not clear and there are many different views on this issue. Usually, weight loss could be seen in a severe level of liver cancer [32]. Apart of many potential underlying variables, which could affect weight changes in liver cancer, two of the major factors are severity and interval of acquiring the disease. Biochemical results of the present study showed promising outcomes. AFP is one of the old but yet the most widely used blood marker tests for liver cancer. High level of AFP among the control group of the present study was similar to previous studies. Many of the studies in the last four decades have shown that AFP was elevated in hepatocarcinogenesis and embryonic carcinomas [33–35]. Beneficial effect of* T. polium* in the present study was supported by previous studies [34, 36]. It was shown that antioxidants can decrease the level of AFP [37, 38]; therefore, phytochemicals or any antioxidant active compounds of* T. polium* which has increased TAS level might affect the AFP level and could express this beneficial effect. Concerning ALP, it has been shown that ALP among liver function tests, in addition to other tumor characters, is an independent factor for disease-free survival and overall survival [39, 40]. Recent studies have suggested that preoperative ALP levels could be utilized to monitor and predict recurrence in high risk HCC patients [41, 42]. Both normal and* T. polium* treated group showed significantly lower serum ALP (*P* < 0.05), which was similar to previous studies [43–45]. Significant elevation of serum AST and ALT activities was seen in a variety of liver conditions, including viral infection, cirrhosis, nonalcoholic steatohepatitis (NASH), drug toxicity, liver tissue degeneration, and necrosis [46]. AST elevations often predominate in patients with cirrhosis and even in liver diseases that typically have increased ALT level [47]. Both normal group and* T. polium* treated group showed significantly lower serum AST and ALT (*P* < 0.05). Beneficial effect of* T. polium* on liver enzymes, including AST and ALT, has been reported previously [43, 45] and the present study supported the previous claims as well. Elevated AST/ALT ratio is clinically accepted as a better marker than assessing individually [48, 49]. Unlike other studies [43], non-significantly higher ratio was found among* T. polium* group. Lack of significant result might be due to the higher standard error of mean in both control and treatment group. Lack of significant result might be due to the higher standard error of mean, among both control and treatment groups.

**Figure 4:**
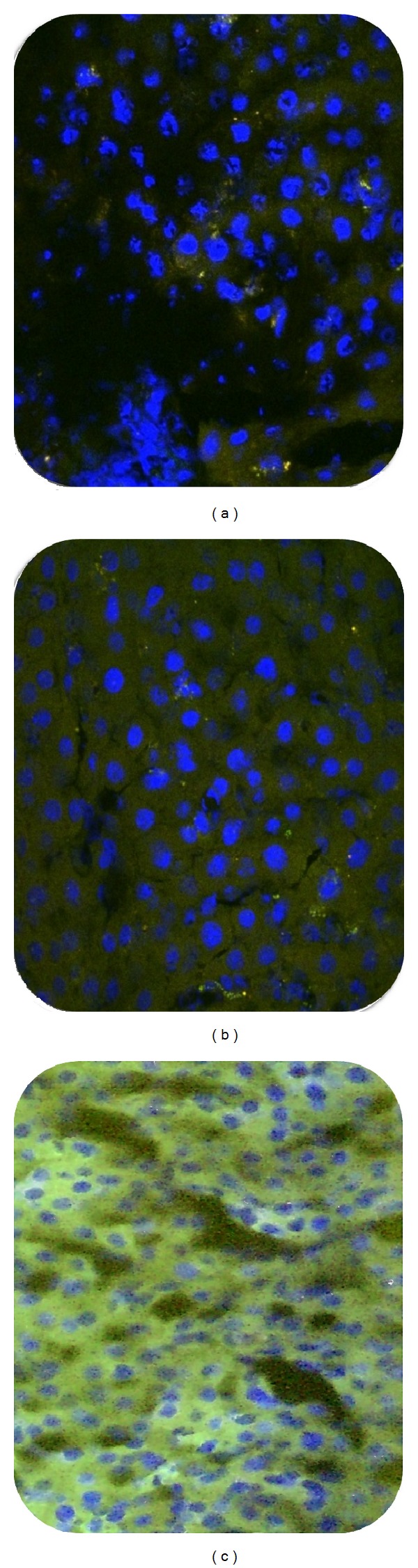
Fluorescent* in situ* hybridization micrograph of lobular region of protein expression of glucocorticoid receptors in the cytoplasm. (a) Normal. (b) Control. (c)* T. polium* treated group. Blue: nuclei with DAPI; green: protein expression of glucocorticoid receptors in cytoplasm. Frozen section, ×600 magnification.

Glucocorticoids (GCs) are frequently used to support patients suffering from various types of cancers. Their key therapeutic role is based on the GC receptor- (GR-) mediated mechanisms that activate cell death; however, this differs depending on the type of cancer [50]. Glucocorticoids prevent prostaglandin synthesis at the level of phospholipase A2 as well as at the level of cyclooxygenase/PGE isomerase (COX-1 and COX-2) [51]. The latter effect is similar to nonsteroidal anti-inflammatory drugs (NSAIDs), which potentiate the anti-inflammatory effect [52, 53]. COX-2-dependent activity is an essential element for cellular and molecular mechanisms of cancer cell motility and invasion. The COX-2 activity also modulates the expression of matrix metalloproteinase (MMP), which may be a part of the molecular mechanism by which COX-2 promotes cell invasion and migration [52]. Many studies on different types of cancer have shown that cyclooxygenase suppression would decrease cancer cells [52–54]. Therefore, cyclooxygenase suppression by glucocorticoids might decrease risk of cancer or control its metastasis [55]. Glucocorticoids inhibit hepatocellular proliferation and modulate the expression of oncogenes and tumor suppressor genes via mechanisms involving the glucocorticoid receptor. Glucocorticoids also produce a receptor-mediated inhibitory effect on both basal and hormone-stimulated expression of a newly discovered family of molecules important for shutting off cytokine action [56] as well as different caspase pathways [57]. Based on the present study,* T. polium* has glucocorticoids stimulation activity, which might have a positive effect on cancer prevention or treatment. The results of confocal microscopy of fluorescent* in situ* hybridization of liver cells helped us to have a better answer for our findings in both light microscopy and biochemical results. As the FISH result in Figure 4 illustrated, high level of glucocorticoid receptor activity was observed in* T. polium* treated group. Therefore, higher activity of GC receptors and higher level of serum CBG, which have been found in the present study, could also explain the possible anticancer or cancer suppressor competences of* T. polium*. Overall, as the present study is the first study in its field, further study would help us to have a better view of the mechanism of action of this herb on glucocorticoids stimulation.

It is well established that the elevated serum GGT activity could be found in diseases of the liver, biliary system, pancreas, and different types of cancers including HCC [58, 59]. In the present study, significantly lower level of GGT was found in* T. polium* treated groupas compared to control group (*P* < 0.05). Both epidemiological and experimental studies found a linkage between hyperhomocysteinemia and a varied range of impaired liver functions like cirrhosis and chronic alcohol consumption [60]. The present results showed significantly higher levels of HCY in the control group and are supported by few studies which have found high levels of HCY in different types of cancer [61] and liver disorders [62]. Recently it has been demonstrated that HCY inhibited hepatocyte proliferation by upregulating protein levels of p53 as well as mRNA and protein levels of p21Cip1 in primary cultured hepatocytes. HCY induced TRB3 expression via endoplasmic reticulum stress pathway, causing Akt dephosphorylation. Knockdown of endogenous TRB3 meaningfully suppressed the inhibitory effect of HCY on cell proliferation and the phosphorylation of Akt. [63]. Significantly lower HCY level in* T. polium* treated group showed vaguely that* T. polium* compounds could possibly suppress tumorigenicity through other possible mechanisms rather than HCY and its mentioned cascade pathway. Unfortunately, like GGT, the present study is the first attempt to observe the effect of this herb on serum level of HCY and further studies are needed to have better insight.

The role of TNF-*α* in liver cancer looks as a mixed blessing. TNF-*α* is a pleiotropic cytokine that can make both cell death and cell proliferation. TNF-*α*-induced cell death is normally blocked by the concurrently activated NF-*κ*B pathway [64]. Deregulated TNF expression within the tumor microenvironment seems to favor malignant cell tissue invasion, migration, and final metastatic formation. On the other side, TNF-*α* clearly possesses antitumor effects not only in preclinical models, but also in the clinical ones [65]. It has been suggested that TNF-*α* plays an important role in the progress of different types of cancer including liver cancer [66]. The role of TNF-*α* has been connected to all steps engaged in tumorigenesis, including cellular transformation, promotion, survival, proliferation, invasion, angiogenesis, and metastasis [67]. Based on the present study, anticancer activity of* T. polium* compounds could possibly suppress TNF-*α* elevation which also might have either direct or indirect beneficial reciprocal effect on other markers.

Animal studies showed that *α*2MG is an imperative novel cytochemical marker to identify hepatocellular preneoplastic and neoplastic lesions, particularly amphophilic cell foci, undetectable by establishing cytochemical markers, and is tightly linked to rat hepatocarcinogenesis [68]. Furthermore, a number of authors have reported upregulation of serum *α*2MG in association with HCC in humans being significantly raised as compared to liver cirrhosis and amoebic liver abscess [69]. In the present study* T. polium* treated group showed significantly lower levels of *α*2MG as compared to control group. It is shown that cancer cells produce and secrete large amounts of *α*2MG, which seems to be linked with their tumorigenicity [70]; therefore,* T. polium* might decrease *α*2MG secretion through cancer tumorigenicity suppression.

**Figure 5:**
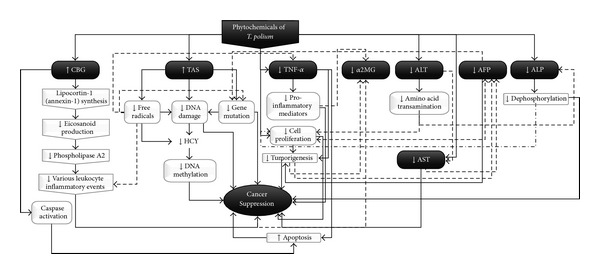
Brief possible beneficial effects of* T. polium* on HCC.

Extremely high level of TAS in treated group could be one of the master keys in revealing possible cancer suppressor capabilities of* T. polium*. It has been shown that excessive reactive oxygen species (ROS) cause oxidative damage to biomolecules and lead to cellular alterations and ultimately tumorigenesis and neoplastic transformation [71]. Therefore, high level of TAS not only could act as excess ROS protector, but also might indirectly affect other cancer markers which have been tested.  Figure 5 illustrates the possible beneficial effects of* T. polium* on HCC.

Lesion score evaluation of rats' liver also showed* T. polium* decoction successfully reduced the score of inflammation or necrosis at the both portal and lobular area as compared to control group (*P* < 0.05), which was comparable with previous anticancer herbal studies [34, 36].

These beneficial effects of* T. polium* could be explained by the significantly high level of flavonoid compounds and antioxidant competency of the* T. polium*. As the present study failed to find both rutin and apigenin as two of the active compounds of* T. polium* anticancer activities [72, 73], in order to find out the active compounds that play major role(s) in producing these effects, and possible molecular mechanisms, further studies are therefore necessary.

## Figures and Tables

**Table 1:** Comparison of the DPPH scavenging activity of *T. Polium* decoction with standards.

Substance	20	40	80	160	IC_50_
	Mean ± SEM (*μ*g/mL)				
*T. Polium *	9.1 ± 0.8^a^	48.4 ± 1.7^a^	83.7 ± 0.5^a^	81.2 ± 1.4^a^	41.5 ± 0.7^a^
*α*-Tocopherols	46.0 ± 0.4^b^	86.6 ± 1.9^b^	91.6 ± 1.2^b^	91.2 ± 3.5^b^	22.4 ± 0.8^b^
Rutin	41.9 ± 0.3^b^	78.7 ± 1.3^b^	80.2 ± 1.7^a^	80.8 ± 1.0^a^	24.5 ± 0.5^b^
BHA	45.41 ± 0.4^b^	81.9 ± 1.2^b^	85.8 ± 0.4^b^	87.0 ± 5.5^a^	22.5 ± 0.6^b^

^ab^Values in the same column with different superscripts are significantly different at *P* < 0.05 based on one-way ANOVA and Duncan's post hoc test.

**Table 2:** Effect of *T. polium *on serum biochemical markers as compared to normal and control groups.

Marker	Normal (*n* = 10)	Control (*n* = 8)	*T. polium *(*n* = 11)
ALP (IU/L)	38.78 ± 1.72^a^	77.77 ± 3.74^b^	52.9 ± 3.8^c^
ALT (U/L)	25.84 ± 1.66^a^	67.20 ± 4.91^b^	40.20 ± 3.37^c^
AST (U/L)	56.62 ± 2.53^a^	156.18 ± 10.64^b^	91.89 ± 5.40^c^
AST/ALT ratio	2.24 ± 0.08	2.47 ± 0.25	2.51 ± 0.31
CBG (*μ*g/mL)	10.74 ± 0.26^a^	11.49 ± 0.35^b^	13.43 ± 0.30^c^
HCY (nmol/mL)	0.57 ± 0.03^a^	1.41 ± 0.14^b^	0.85 ± 0.08^c^
TNF-*α* (pg/mL)	24.03 ± 1.00^a^	49.08 ± 1.12^b^	32.98 ± 1.17^c^
*α*2MG (ng/mL)	0.71 ± 0.04^a^	1.42 ± 0.09^b^	0.91 ± 0.07^c^
AFP (pg/mL)	47.51 ± 1.05^a^	101.85 ± 2.86^b^	63.52 ± 3.70^c^
TAS (mmol/L)	9.84 ± 0.35^a^	1.09 ± 0.16^b^	10.48 ± 0.06^c^
GGT (mU/mL)	0.68 ± 0.01^a^	1.14 ± 0.04^b^	0.77 ± 0.01^c^

^abc^Values in the same row with different superscripts are significantly different at *P* < 0.05 based on one-way ANOVA and Duncan's post hoc test. Data were presented as mean ± SEM.
